# Is Natural Population of *Candida tropicalis* Sexual, Parasexual, and/or Asexual?

**DOI:** 10.3389/fcimb.2021.751676

**Published:** 2021-10-25

**Authors:** Jianping Xu

**Affiliations:** ^1^ Institute of Bast Fiber Crops and Center of Southern Economic Crops, Chinese Academy of Agricultural Sciences, Changsha, China; ^2^ Department of Biology, McMaster University, Hamilton, ON, Canada

**Keywords:** mode of reproduction, mutation accumulation, loss of heterozygosity, signatures of recombination, ploidy

## Abstract

*Candida tropicalis* is one of the most common opportunistic yeast pathogens of humans, especially prevalent in tropical and subtropical regions. This yeast has broad ecological distributions, can be found in both terrestrial and aquatic ecosystems, including being associated with a diversity of trees, animals, and humans. Evolutionary theory predicts that organisms thriving in diverse ecological niches likely have efficient mechanisms to generate genetic diversity in nature. Indeed, abundant genetic variations have been reported in natural populations (both environmental and clinical) of *C. tropicalis*. However, at present, our understanding on how genetic diversity is generated in natural *C. tropicalis* population remains controversial. In this paper, I review the current understanding on the potential modes of reproduction in *C. tropicalis*. I describe expectations of the three modes of reproduction (sexual, parasexual, and asexual) and compare them with the observed genotypic variations in natural populations. Though sexual and parasexual reproduction cannot be excluded, the analyses suggest asexual reproduction alone could explain all the observations reported so far. The results here have implications for understanding the evolution and epidemiology of *C. tropicalis* and other related human fungal pathogens.

## Introduction

*Candida tropicalis* is a common opportunistic yeast pathogen, capable of causing both superficial and systemic infections in humans (Zuza-Alves et al., 2017). *C. tropicalis* was first isolated from a patient with fungal bronchitis in 1910 and was initially named *Oidium tropicale* (Castellani, 1912). Since then, this yeast has been found in diverse ecological niches such as in the soil, aquatic environments (both fresh water and marine ecosystems), a diversity of trees and plant debris, as well as from humans and other animals (e.g., Odds, 1988; Carvalho et al., 2014; Brilhante et al., 2015; Zuza-Alves et al., 2017; Aljohani et al., 2018). Similar to its broad ecological distribution, *C. tropicalis* is also geographically widely distributed, particularly prevalent in tropical and subtropical regions such as Southeast Asia, Africa, and Central and South America (Zuza-Alves et al., 2017; Aljohani et al., 2018; Wu et al., 2019; Samarasinghe et al., 2021). In humans, *C. tropicalis* has been isolated from diverse body sites, from our skin to the oral cavity, the gastrointestinal tract, and the urogenital systems in many healthy hosts, and in patients with systemic infections including the circulatory system and other typically sterile body sites (Zuza-Alves et al., 2017; Wu et al., 2019). Among patients with systemic candidiasis, *C. tropicalis* is commonly reported as the second- or third-most common causative agent, especially in those with hematological malignancies (Zuza-Alves et al., 2017). Bloodstream infections by *C. tropicalis* are associated with high mortality rates, ranging from 41–61% (e.g., Muñoz et al., 2011).

Triazole antifungals are among the first-line drugs for treating candidiasis. Importantly, compared to several other closely related species such as *Candida albicans, Candida dubliniensis*, and *Candida parapsilosis*, *C. tropicalis* typically has a much higher frequency of resistance to triazoles, up to 36% in some populations (e.g., Fan et al., 2017; Zuza-Alves et al., 2017). Patients infected with triazole-resistant *C. tropicalis* typically have higher mortality rates than those infected with triazole-susceptible strains. Interestingly, geographic regions differ in the frequencies of triazole-resistant *C. tropicalis*, with those from the Asia-Pacific regions showing a higher frequency of resistance to fluconazole than those from other regions (Pfaller et al., 2019). Since genotypic variation is an important component of pathogen populations’ adaptation to diverse ecological niches and environmental stresses, it’s critical to understand the mechanisms for the origin, maintenance, and spread of genetic variation in pathogen populations. Such knowledge could help develop better prevention and control measures against pathogen infections, including those by *C. tropicalis*. The objectives of this paper are to provide a critical review on the potential mechanisms for how genetic variations are generated in *C. tropicalis*, with a focus on the potential modes of reproduction.

Below I first briefly describe the genetic and genomic features of *C. tropicalis*, followed by a general overview of how genetic variations are generated and maintained in cellular organisms. I then describe the expectations of three modes of reproduction (sexual, parasexual, and asexual reproductions), summarize the observed population genetic variation and evidence for recombination in *C. tropicalis*, and discuss how asexual reproduction alone could explain the observed genetic variation reported so far in natural populations of *C. tropicalis*.

## Genetic and Genomic Features of *C. tropicalis*

*Candida tropicalis* is a diploid yeast. It is a member of the CUG-Ser1 clade of ascomycete fungi that include *C. albicans, C. parapsilosis, Candida auris, Debaryomyces hansenii (*syn*. Candida famata)*, and *Clavispora lusitaniae (*syn*. Candida lusitaniae)* in which the CUG codon is translated as serine instead of leucine in the standard genetic code (Krassowski et al., 2018). The genome of *C. tropicalis* was first sequenced in 2009 (strain MYA-3404), revealing an estimated genome of 14.6 million base pairs (Mbp) that contains 6,254 protein-encoding genes, 432 non-protein coding genes, and one pseudogene for a total of 6,687 genes (Butler et al., 2009; Guin et al., 2020). The *C. tropicalis* genome contains abundant genes coding for virulence factors such as those involved in adhesion to host tissues, morphogenesis, phenotypic switching, biofilm formation, and those with proteinase, phospholipase, and hemolytic activities. The whole genome sequence has been assembled into one mitochondrial scaffold and seven pairs of chromosomes in the nuclear genome (Guin et al., 2020). Interestingly, early research revealed that the *C. tropicalis* genome consisted of 12 different-sized chromosomes, with notable chromosomal length polymorphisms among the three strains analyzed by pulse field gel electrophoresis (Doi et al., 1992). These genomic features suggest that chromosomal rearrangements such as duplications and translocations are likely common in natural populations of *C. tropicalis*. Chromosomal rearrangements such as inversion and translocation can suppress crossing-overs, accelerate mutation accumulation and sequence divergence between homologous chromosomes, and impact the genetic variations within and among individual strains.

## Sources of Genetic Variation

In all organisms, genetic variations can be expressed at different levels, e.g., at the whole genome, individual chromosome, individual gene/DNA fragment, and individual nucleotide levels. At the whole genome level, virtually all unrelated individuals of a species would be genetically different from each other. Such genetic differences could be shown as whole-genome nucleotide sequence differences as well as chromosome structural polymorphisms such as chromosomal segment or whole chromosome duplications and deletions (aneuploidy), and chromosomal translocations. At the individual chromosome level, differences among homologous chromosomes from different individuals can be observed as whole chromosomal nucleotide sequence differences. Such chromosomal level differences may be caused by insertions, deletions, duplications, transpositions, inversions, and base substitutions. At the individual gene level, differences between alleles within the same strain (in diploids and polyploids) and among strains are primarily shown as differences in nucleotide sequences which can include small insertions and deletions as well as base substitutions. Lastly, at the individual nucleotide level, polymorphisms are shown as base substitutions and single nucleotide insertions and deletions, collectively called single nucleotide polymorphisms (SNPs).

At present, the rates of different types of genetic changes in *C. tropicalis* (and indeed most other organisms) in natural environments are not known. In the model yeast *Saccharomyces cerevisiae*, genome sequencing of 145 mutation accumulation (MA) lines that went through 100 severe population bottlenecks each, with each bottleneck separated by about 20 mitotic divisions (i.e., asexual or clonal reproduction), revealed 867 novel single base substitutions, 26 small insertion/deletions (indels) of under 50 bp each, three copy number variants, and 31 whole-chromosome copy-number changes (29 were duplications and two were losses) (Zhu et al., 2014). These observed changes led to an estimated genome-wide single-base substitution rate at 1.67 ± 0.04 × 10^−10^ per base per generation; an indel mutation rate of 5.03 ± 0.99 × 10^−12^ per base per generation; a whole-chromosome duplication rate of 9.7 ± 1.8 × 10^−5^ events per diploid genome per generation; and a whole chromosome loss rate of 0.7 ± 0.04 × 10^−5^ events per diploid genome per generation (Zhu et al., 2014). As expected, due to the frequent severe population bottlenecks, the observed mutations followed a neutral mutation model. Among these novel mutations, the observed indels included one tandem-repeat insertion and one tandem-repeat deletion, all but one of the 26 indels occurred near simple sequence repeats, indicating sequence repeat regions as indel mutation hotspots (Zhu et al., 2014). None of the 145 MA lines shared any single nucleotide substitutions or indels. In contrast, shared chromosomal duplications were common among the MA lines. The observed nucleotide substitution rate was overall similar (within ±10x) to those estimated for other organisms using experimental evolution approaches, such as the model bacterium *Escherichia coli*, a common yeast pathogen *C. albicans*, the model flowering plant *Arabidopsis thaliana*, the fruit fly *Drosophila melanogaster*, and mouse (Xu, 2012). It’s highly likely that *C. tropicalis* has a similar mutation spectrum and rate (within ±10x) as *S. cerevisiae*. However, the generation time of *C. tropicalis* in nature is not known and will likely differ among geographic regions and ecological niches where individual strains and populations reside. Thus, there is significant uncertainty in the expected number of mutations that can be accumulated in a given time period among the geographic and ecological populations of *C. tropicalis*.

Aside from mutation, another source of genetic variation among strains in natural populations is recombination. In diploid organisms such as *C. tropicalis*, recombination can happen during asexual, parasexual, and/or (potentially) sexual reproduction. For example, mitotic recombination can happen for all organisms using the three different modes of reproduction while meiotic recombination happens only during meiosis I during sexual reproduction. In addition, both chromosomal reassortment and crossing-over could happen during all three modes of reproduction to contribute to the generation of new genotypes in populations.

In contrast to mutation and recombination that can contribute to increases in overall genotypic diversity and genetic variations in populations, selection and genetic drift can reduce genetic variation. Several factors can influence mutation, recombination, selection, and drift in populations, including population size, modes of reproduction, environmental niche heterogeneity, and other biotic and abiotic interacting factors such as the presence/absence of stresses and/or mutagens. In the next section, I will focus on the potential impacts and expectations of each of the three modes of reproduction on genetic variation in *C. tropicalis*.

## Expectations From Different Modes of Reproduction in Nature

Like most yeasts, *C. tropicalis* can reproduce efficiently asexually through budding. Indeed, *C. tropicalis* can grow rapidly on many artificial media through mitosis and asexual budding. Prior to 2011, *C. tropicalis* was considered an asexual species. However, it is now known that *C. tropicalis* can mate and have a parasexual cycle in artificial laboratory environments (Porman et al., 2011; Seervai et al., 2013). However, the occurrence and importance of parasexuality in natural *C. tropicalis* populations is not known. At present, despite the successes in laboratory mating involving either the same or opposite mating types, meiosis and ascospore production have never been observed in *C. tropicalis* either in lab setting or in nature. It should be noted though that the absence of such observation does not mean that sexual reproduction is completely absent in *C. tropicalis* in nature. Indeed, as has been shown recently, under appropriate conditions, several traditionally considered “asexual” fungi have shown capabilities for sexual reproduction (Ni et al., 2011; Nieuwenhuis and James, 2016). Thus, despite our lack of success for inducing sexual reproduction for *C. tropicalis* in the lab, such a condition might exist in nature for *C. tropicalis*. Below, I outline the genetic expectations of sexual, parasexual, and asexual reproductions in natural populations of *C. tropicalis*.

### Expectations of (a Hypothetical) Sexual Reproduction in *C. tropicalis*

Sexual reproduction in typical eukaryotes involves mating between cells/gametes of opposite sex, followed by growth of the mating product, and meiosis to generate sexual progeny, restoring the ploidy level of the parental cells/gametes. During mating, both sexual partners contribute equal nuclear genetic materials to the offspring while typically only one parent contributes the mitochondrial genome. During meiosis, the nuclear genes from the two parental sources are recombined to produce genetically diverse gametes, with each gamete containing approximately equal proportions of nuclear DNA from the two original parents.

In the case of *C. tropicalis*, sexual reproduction, if it happened in nature, would likely involve one of two forms. In the first, diploid strains that are homozygous for the *MTL* **a** and *MTL* **α** idiomorphs mate to form tetraploids. Tetraploid cells are capable of mitotic division and clonal reproduction, same as the diploid parental cells. If sexual reproduction existed, the tetraploid cells of *C. tropicalis* would undergo meiosis and their sexual progeny would be diploid and recombinants. Further, if the diploid sexual progeny were homozygous at the mating type locus (about 50% of the progeny, assuming random chromosome segregation during meiosis), those with different mating types could be capable of mating again to repeat the sexual cycle. In the second form, natural diploid strains that are heterozygous at the mating type locus could undergo meiosis to generate a diversity of recombinant haploid progeny inheriting either the *MAT* **a** or *MAT* **α** mating type which then could mate with cells of opposite mating types to restore diploidy. In both forms of sexual reproduction, the progeny population would show random associations between genetic markers located on different chromosomes while those on the same chromosomes would show different degrees of statistical association (i.e., genetic linkage) depending on their distance from each other on the chromosome. Segregation, random assortment, and recombination of genetic markers should all follow the Mendelian laws of inheritance. Thus, in natural populations of *C. tropicalis*, if sexual reproduction happened (even occasionally), we would expect to see evidence for two forms of allelic associations: (i) Hardy-Weinberg equilibrium among alleles from the same locus; and (ii) random association among alleles at different loci (i.e., linkage/gametic equilibrium and genotypic equilibrium), including loci located on different chromosomes (due to chromosomal random reassortment during meiosis) or on the same chromosome (due to crossing-over between homologous chromosomes during meiosis I) (**Table 1**).

**Table 1:** Population genetic expectations of different modes of reproduction.

Signatures of recombination	Sexual reproduction	Parasexual reproduction	Asexual reproduction^2^	Note
Hardy-Weinberg equilibrium	++++^1^	++	+	Applicable to diploid and higher ploidy organisms
Phylogenetic incompatibility	++++	++	+	Also called “four-gamete test”.
Linkage/gametic equilibrium	++++	++	+	Applies more easily to haploid organisms than to diploids and polyploids where haplotype phasing is needed first.
Genotypic equilibrium	++++	++	+	Applicable to all ploidy organisms.
Haplotype or allele number > number of SNP sites + 1	++	+	+	Commonly used to identify evidence of intragenic recombination.
Gene genealogy incongruence	++++	++	±	Also called “incongruence length difference” test. Not used in *C. tropicalis* yet.

^1,^ ++++, very common; ++, somewhat common; +, infrequent.

^2,^ please refer to **Figure 1** for processes to generate signatures of recombination.

Recombination can happen in all three modes of reproduction.

### Expectations of Parasexual Reproduction in *C. tropicalis*

In *C. tropicalis*, a parasexual cycle has been described under laboratory conditions (Porman et al., 2011; Seervai et al., 2013). Specifically, during a parasexual cycle, diploid cells that are homozygous for either the *MTL*a or *MTL*α idiomorphs undergo phenotypic switching from white to the opaque state and then mate (Porman et al., 2011; Xie et al., 2012). The mating products are tetraploid and heterozygous at the mating type locus (*MTL*a/α). These tetraploid cells are capable of indefinite clonal reproduction. However, in one estimate, these tetraploid *MTL*a/α cells revert to the diploid state very rapidly (after ~240 mitotic generations, Seervai et al., 2013) through a concerted chromosome loss process. This process results in diploid cells that are either heterozygous *MTL*a/α or homozygous for either the *MTL*a or *MTL*α idiomorph. While the heterozygous *MTL*a/α cells are unable to mate, the homozygous *MTL*a and *MTL*α cells are capable of mating with each other again to resume the parasexual cycle. Aside from diploid *MTL*a x diploid *MTL*α mating, same-sex mating (i.e., mating between two cells homozygous for the same mating type) has also been observed in *C. tropicalis*. However, same-sex mating was only observed in the presence of the pheromone from the opposite mating type (Du et al., 2018). Among laboratory strains from the parasexual cycle, only diploid and tetraploid have been reported, consistent with concerted rapid chromosomal loss throughout the whole genome (Seervai et al., 2013). During the reversion from tetraploid to diploid, though the occurrence and rate of mitotic recombination have not been examined in *C. tropicalis*, but as has been demonstrated in *C. albicans* (Forche et al., 2011), mitotic crossing-over could happen and chromosome loss might be random, both of which could generate recombinant genotypes for markers located on either the same or different chromosomes. Interestingly, under stress conditions such as thermal shock, exposure to UV light, and growth in l-sorbose or d-arabinose as the only carbon source, the rate of chromosome loss and the frequencies of diploid cells increase significantly in experimental populations of a synthesized tetraploid strain of *C. tropicalis* (Seervai et al., 2013). Such a stress-associated chromosome loss likely represents an adaptive response (Morrow and Fraser, 2013).

Parasexual reproduction, if it happened in natural populations, would lead to the following expectations. First, as described above, mating between diploid strains should produce tetraploid strains and since tetraploid strains are capable of clonal reproduction, tetraploids and aneuploids (> 2N, derived from the spontaneous loss of chromosomes during diploidization from tetraploids) should be present in nature. Second, since chromosomal losses may be random, genetic polymorphisms on different chromosomes may be lost independently during reversion from tetraploidy to diploidy. Consequently, there could be random associations among alleles and genotypes between loci located on different chromosomes. Third, though crossing-over has not been demonstrated during parasexual reproduction in *C. tropicalis* in the lab, likely due to limited genetic analysis, if it happened in nature, we should expect similar evidence for linkage/gametic equilibrium and genotypic equilibrium between loci on the same chromosomes in natural populations. In addition, the degree of statistical association among alleles for loci on the same chromosomes should be higher than those located on different chromosomes.

As can be seen from the above, while there might be quantitative differences in the frequencies of aneuploidy and in the degrees of associations among alleles at the same or different loci, at the population level, the expectations listed above for parasexual reproduction in nature are similar to those expected for sexual reproduction (**Table 1**).

### Expectations of Asexual Reproduction in *C. tropicalis*

Similar to other microorganisms (Xu, 2009; Taylor et al., 2015), asexual reproduction is likely the most common mode of reproduction for *C. tropicalis* in nature. Asexual reproduction in *C. tropicalis* is accomplished through mitosis and budding. While asexual reproduction is typically considered a stringent mechanism for faithful transmission of the entire genetic materials from one generation to the next, however, like in parasexual and sexual reproductions, different types of mutations can occur and accumulate during asexual reproduction (e.g., Zhu et al., 2014). In the diploid genome of *C. tropicalis*, during asexual reproduction, the two homologs of each of the seven chromosome pairs could accumulate different mutations, including different base substitutions, indels, copy number variants, and chromosomal losses and duplications (due to mitotic non-disjunction). Furthermore, aneuploids such as those with 2N-1 and 2N+1 (derived due to mitotic non-disjunction) can return to diploids through chromosomal duplication and chromosomal loss, respectively. The accumulation of mutations without mitotic crossing-over or chromosomal loss would lead to the divergence between homologous chromosomes during asexual reproduction within each strain and clonal lineage. In contrast, chromosomal loss would lead to reduction or even complete loss of heterozygosity within individual chromosomes, strains and lineages. The rates of mutation and chromosomal loss would determine the rate of mutation accumulation within individual strains and populations. Similarly, the inclusion of mitotic crossing-overs coupled with chromosome loss would impact the heterozygosity distribution among different regions within each chromosomal pair. During asexual reproduction, with chromosomal loss, a heterozygous individual, e.g., a SNP with A/G nucleotide combinations, could generate two homozygous genotypes (i.e., A/A and G/G). In combination with the original parental heterozygote A/G genotype in the population, the asexual reproduction of three genotypes A/A, G/G and A/G could lead to their frequencies in the populations approaching Hardy-Weinberg equilibrium. Furthermore, through mitotic crossing-over and random chromosomal loss, alleles and genotypes at different loci (located either on the same or different chromosomes) could approach linkage/gametic equilibrium and genotypic equilibrium (**Table 1**).

The above analyses indicated that, in essence, the signatures of recombination (Hardy-Weinberg equilibrium, linkage/gametic equilibrium, and genotypic equilibrium etc.) commonly used for inferring recombination in natural microbial populations could be achieved through all three modes of reproduction in *C. tropicalis* (**Table 1**). In the section below, I briefly review our current understanding of the population genetic structure of *C. tropicalis* in nature and compare the observed genetic variations with expectations described in this section, with an emphasis on signatures of recombination.

## Evidence for Clonality and Recombination in Natural *C. tropicalis* Populations

Over the past four decades, a variety of genetic markers have been used to analyze strains and populations of human pathogenic fungi (Hong et al., 2021), including for *C. tropicalis* (e.g., Tavanti et al., 2005; O’Brien et al., 2021). Most of these genetic markers are based on variations in DNA sequences. Since 2005, multilocus sequence typing (MLST) using sequence variations in fragments of six unrelated genes has been the dominant method for studying genetic variations within and among strains as well as for inferring population genetic and epidemiological patterns of *C. tropicalis* across different scales, from local to regional, national, continental, and global scales (Tavanti et al., 2005; Jacobsen et al., 2008; Wu et al., 2019). More recently, whole genome sequencing has been used to identify the patterns of genomic variation and to study the origins of certain strains (O’Brien et al., 2021). Below I summarize evidence for clonality (i.e., asexual reproduction) and recombination in nature based on data from MLST and whole-genome sequencing.

In 2008, Jacobsen et al. analyzed MLST data at six loci for 262 isolates of *C. tropicalis* from a few geographic regions (primarily from the UK and Taiwan). Their MLST data separated the 262 isolates into 242 multilocus sequence types. More recently, Wu et al. (2019) analyzed 876 isolates of *C. tropicalis* in the MLST database and found that 280 nucleotide sites out of the total 2677 aligned nucleotide sites concatenated based on the six sequenced gene fragments were polymorphic. These 280 SNPs represented 10.45% of the 2677 total aligned sites and separated the 876 isolates into 633 multilocus sequence types. Among the 633 multilocus sequence types, 93 were shared by strains from within and between different local, regional, national, and continental samples (Wu et al., 2019). Given the large number of genotypes at each locus [an average of 81.67 and a range of 38-150 sequence types/locus; Wu et al. (2019)], the identification of strains from different sources and geographic areas but with the same multilocus sequence types are consistent with asexual reproduction and clonal dispersal across geographic scales in natural populations of *C. tropicalis*.

To test whether there was evidence for recombination, Jacobsen et al. (2008) compared the observed genetic variations with several expectations of either no recombination or recombination in their sample. First, they compared the relationships between the number of SNPs and the number of haplotypes in the population. Specifically, they reasoned that if there was no recombination between haplotypes, nor any recurrent or reverse mutation, then *n* SNPs in any gene fragment would be expected to result in *n+1* haplotypes for that gene fragment in the population. Consequently, the number of haplotypes greater than *n+1* would be suggested as evidence for recombination among SNP sites within individual gene fragments (**Table 1**). To test for this prediction, the authors chose 57 isolates representing the broad 242 MLST genotypes and inferred the most likely haplotypes in the population for each of the six genes, using the program FastPHASE (Scheet and Stephens, 2006). Based on this analysis, two of the six loci, *MDR1* and *XYR1*, had far more inferred haplotypes than expected based on *n+1*. They concluded that recombination had happened among SNP sites within each of the two gene fragments, with multiple recombination events likely involved in generating the inferred number of haplotypes at the *XYR1* locus (Jacobsen et al., 2008).

Second, they conducted phylogenetic incompatibility test or the commonly called “four gamete test” and found evidence of recombination among SNP sites within all six gene fragments, including the four gene fragments with lower than *n+1* haplotypes. Third, among the total 169 SNPs (out of the 2677 nucleotide sites for the six sequenced gene fragments) in their sample, they chose 22 SNPs where the minor allele frequency was at least 15% to examine whether their genotype distributions fitted Hardy-Weinberg equilibrium. Among these 22 SNPs, six SNPs located in three genes showed no significant deviation from Hardy–Weinberg equilibrium. Lastly, their analysis of all 231 pairwise combinations of the 22 SNPs showed that 93 pairs were not significantly different from linkage/gametic equilibrium, including SNP pairs located within the same gene and between genes. The above results were all consistent with some form of recombination in natural populations of *C. tropicalis* (Jacobsen et al., 2008).

Evidence for recombination in *C. tropicalis* natural populations has also been reported from the analyses of whole-genome sequence data. For example, within the genome of the *C. tropicalis* model strain MYA-3404, Guin et al. (2020) detected that a long stretch on the left arm of Chromosome R had very low SNPs and indels while the remaining part of this chromosome showed high and comparable heterozygosity distributions as other six chromosomes. This observation suggested that a mitotic crossing-over followed by chromosome loss had likely happened between the two homologs of Chromosome R (Guin et al., 2020). Similarly, differences in heterozygosity among chromosomes and chromosomal segments were observed within many of the 77 natural strains of *C. tropicalis* recently published by O’Brien et al. (2021). Furthermore, genome-wide heterozygosity varied widely among the 77 strains, with a range of 0.2% to 4.9%. The authors attributed the low observed heterozygosity in some strains to chromosome loss and followed by duplication while strains with high levels of heterozygosity were attributed to hybridizations (O’Brien et al., 2021).

## Potential Features Distinguishing Asexual Reproduction From Parasexual/Sexual Reproductions in *C. tropicalis*

As described above, all three modes of reproduction could generate recombinant genotypes that might approach Hardy-Weinberg equilibrium, linkage/gametic equilibrium, and genotypic equilibrium in diploid organisms such as *C. tropicalis* (**Table 1**). However, there are several potential features that can help distinguish asexual reproduction from both parasexual and sexual reproduction in *C. tropicalis*. Below I describe and discuss these features.

The first is that for both parasexual and sexual reproduction, an intermediate mating product in the form of a tetraploid will likely be generated. So far, no tetraploid strain of *C. tropicalis* is known from nature. This is perhaps understandable. In the laboratory setting, the *C. tropicalis* tetraploids formed from mating reverted to diploidy very quickly, at less than 240 mitotic generations for one tetraploid (Seervai et al., 2013). However, triploid and aneuploidy (>2N) has been reported and these could potentially represent intermediate products during the reversion from tetraploid to diploid during the parasexual cycle. Specifically, based on observed allele frequencies within individual strains, among the 77 sequenced strains of *C. tropicalis*, one (ct66) was inferred as a triploid; one (ct26) appeared to be an octaploid; and four strains were single-chromosome aneuploids (strains ct06 and ct18 each had three copies of scaffold 8, and strains ct14 and ct15 both had three copies of scaffold 4) (O’Brien et al., 2021). However, as shown in both *C. albicans* and *S. cerevisiae* (e.g., Forche et al., 2011; Zhu et al., 2014), triploidy and aneuploidy (>2N) could be generated during asexual reproduction, with aneuploidy generated at a relatively high frequency, especially under stress conditions (Morrow and Fraser, 2013). Thus, the observed triploidy and aneuploidy alone in these *C. tropicalis* strains cannot be used as a signature of parasexual or sexual reproduction. Instead, they could also be derived frequently during asexual reproduction, as an adaptive mechanism.

The second potential feature for distinguishing asexual reproduction from parasexual/sexual reproductions is based on heterozygosity patterns of the triploids and aneuploids. Specifically, triploids and aneuploids formed from asexual reproduction would involve the duplication of existing chromosomes or subsets of chromosomes through mitotic non-disjunction and likely represents a short-term adaptation due to environmental stress (and reverts to diploidy in the absence of such stress) (Morrow and Fraser, 2013). Consequently, a maximum of two chromosome-level haplotypes should be found, including a maximum of two nucleotide bases at each nucleotide site throughout the chromosome. In contrast, if the aneuploids were generated through mating during parasexual or sexual reproduction, the chromosome(s) with three copies in the genome would likely all be genetically different from each other. At present, there is no sequenced genome of triploid or aneuploid strains with assemblies at the individual chromosomal level to investigate the possibility of three or more genetically distinct homologous chromosomes within an individual strain. However, based on the MLST data, none of the 876 strains were found to have three nucleotides at any of the 280 SNP sites within the six sequenced gene fragments (Wu et al., 2019). Among the 280 SNP sites (out of the total of 2677 aligned nucleotide sites) in the global population of 876 strains, 13 sites had three nucleotide bases each in the total population but none of the 876 strains had all three nucleotide bases within its genome at any of these 13 SNP sites (Wu et al., 2019). Even if a tri-allelic SNP site were found within a strain, the possibility of gene duplication followed by mutation to a new base at exactly the same nucleotide site in an asexual diploid should be ruled out before the hypothesis for a parasexual/sexual mating is confirmed. Furthermore, among the 77 strains with whole-genome sequences, none showed evidence of chromosome-wide SNP sites with three different nucleotide bases, including the strain with presumed octoploidy (O’Brien et al., 2021).

So far, the strongest evidence that mating might have occurred in natural populations of *C. tropicalis* was the finding of six potential hybrids with unusually high levels of heterozygosity. Specifically, among the 77 isolates sequenced by O’Brien et al. (2021), six were found to have about 10 times the heterozygosity (3.6–4.9% heterozygosity including SNPs and indels) as the remaining 71 isolates (0.2–0.6% heterozygosity; however, one strain (ct20) was very homozygous, had 0.084% heterozygous sites). While hybrid origin could be responsible for these six isolates with high level genome-wide heterozygosity, as suggested by the authors, an alternative possibility have not been ruled out. Specifically, the high-level intra-strain heterozygosity could be generated from mutation accumulation through prolonged asexual reproduction but with limited or no loss of heterozygosity from chromosomal loss events. Indeed, as demonstrated by O’Brien et al. (2021), phylogenetic analysis showed that the six highly heterozygous isolates were extremely divergent both from each other and from other 71 isolates, consistent with their long-term independent accumulation of spontaneous mutations. Interestingly, the presence of evolutionary divergent strains each with a high level of intragenomic heterozygosity has been used as a key piece of evidence for ancient asexuality in several groups of organisms, including the bdelloid rotifers (Welch and Meselson, 2000). Furthermore, in the model sequenced strain MYA-343 of *C. tropicalis*, there were multiple segments across each of the seven chromosome pairs showing a high-level heterozygosity (up to 8.4% heterozygosity in a number of chromosomal regions) (Guin et al., 2020), similar to those for these six putative hybrids observed by O’Brien et al. (2021). Taken together, while the hybrid origin(s) of these six strains are possible and likely (through either sexual or parasexual mating of genetically divergent strains, as suggested by O’Brien et al., 2021), the alternative of (relatively) ancient asexual origins has not been excluded for these six putative hybrids.

While all three modes of reproduction could explain the high heterozygosity within the whole genome or parts of a genome in strains of *C. tropicalis*, all three modes of reproduction could also contribute to low heterozygosity within individual strains of *C. tropicalis*. As described above, low heterozygosity was observed at the whole-genome level in strain ct20 (O’Brien et al., 2021) and parts of the genomes in many strains (Guin et al., 2020; O’Brien et al., 2021). Here, inbreeding through sexual or parasexual reproduction process could generate the low genome-wide heterozygosity observed for strain ct20. However, two asexual reproduction mechanisms could also contribute to low heterozygosity at either the whole-genome level or in parts of the genome. The first is through mitotic crossing-over and chromosomal loss described above, followed by duplication of the remaining chromosomal homolog. The second is through break-induced replication (BIR) during clonal reproduction. BIR is a nonreciprocal, recombination-dependent replication process that can effectively repair broken chromosomes, using homologous chromosomal segments as template for the repair (Kraus et al., 2001). Chromosome breaks can be generated spontaneously but the frequency can increase significantly due to environmental stresses such as ultraviolet light irradiation. Both asexual mechanisms could reduce heterozygosity of the affected whole chromosome or chromosomal segments to zero. After the loss of heterozygosity, continued asexual reproduction would gradually begin to restore heterozygosity through mutation accumulation within these chromosomes/chromosomal segments. Thus, while low heterozygosity could be generated through inbreeding either sexually or parasexually, asexual reproduction involving mitotic recombination, chromosome loss, and/or BIR could also generate the observed heterozygosity patterns.

## A General Model of Asexual Reproduction to Generate Genetic Diversity in *C. tropicalis*

**Figure 1:**
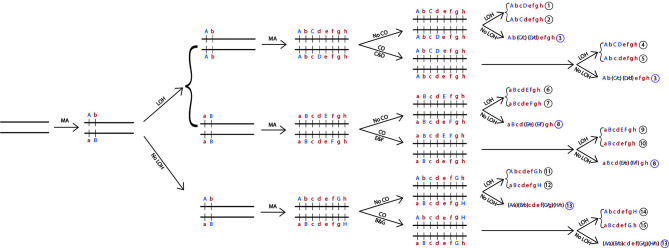
Generation of recombinant genotypes and heterozygosity variations among strains of *Candida tropicalis* through asexual reproduction. Shown here is a single pair of homologous chromosomes completely identical to each other at the beginning and how three processes introduced during asexual reproduction at various stages can lead to the generation of a diversity of recombinant genotypes and heterozygosity patterns among their asexual progenies. The three processes are mutation accumulation (MA), loss of heterozygosity (LOH) through chromosomal loss, and mitotic crossing-over (CO) between different SNP pairs (C & D; E & F; and B & G). The asexual reproduction shown here generated 12 haplotypes and 15 composite diploid genotypes for this chromosome. At each of the eight SNP sites, both homozygous and heterozygous genotypes are found. In addition, there are six recombinant haplotypes 4, 5, 8, 9, 14, and 15. The capital letters A, B, C, D, E, F, G, and H indicate the newly accumulated mutated nucleotides while small letters a, b, c, d, e, f, g, and h indicate the ancestral nucleotides.

Below I further illustrate how asexual reproduction with occasional mitotic recombination and loss of heterozygosity could have generated the observed population genetic variation patterns described so far. In this hypothetical example (**Figure 1**), from a single ancestral strain, a homozygous chromosome pair starts to accumulate spontaneous mutations, with different mutations accumulated among different clonal descendants during asexual reproduction. Here, a total of eight nucleotide substitutions are introduced at eight random sites on this chromosomal pair. The mutated nucleotides are labelled with capital letters A to H. During this time, three single mitotic recombination events through crossing-overs were introduced between different regions of the chromosome. Furthermore, several LOH events (assuming whole-chromosome loss here) were introduced, representing seven reciprocal chromosomal losses or 14 non-reciprocal chromosomal losses. Together, these events would lead to the formation of 15 diploid chromosome sequence types, composed of 12 chromosomal haplotypes, with some strains being more heterozygous than others for this chromosome in the population (**Figure 1**).

In this example, at each of the eight SNP sites, there would be all three possible genotypes: one heterozygous and two homozygous. Depending on their relative fitness and sampling efforts, the proportions of the three genotypes at each site (or most sites) could approach Hardy-Weinberg equilibrium in the population. In addition, a mitotic crossing-over between any two SNP sites on the same homologous chromosomal pair followed by chromosomal loss would result in strains in the population showing evidence of phylogenetic incompatibility and potential linkage equilibrium between the pair of SNP sites.

How likely is the above proposed model? At present, the rates of nucleotide substitution, mitotic crossing-over, and chromosomal loss in *C. tropicalis* are not known. However, in *C. albicans* and *S. cerevisiae*, there have been experimental estimates of all three rates under laboratory conditions. *Candida tropicalis* is in the same Order Saccharomycetales as *C. albicans* and *S. cerevisiae* and all three are naturally diploids. Thus, similar rates as those estimated in *C. albicans* and *S. cerevisiae* may exist in *C. tropicalis* as well. Specifically, in *S. cerevisiae*, the genome-wide single-nucleotide substitution rate was estimated at ~1.67 × 10^−10^ per base per generation; a mitotic crossing-over rate at ~6.2 x 10^-5^ per chromosome per generation; and a whole-chromosome loss rate of ~0.7 × 10^−5^ events per diploid genome per generation (Zhu et al., 2014). Thus, in *S. cerevisiae*, with a genome size of 12 million base pairs (Mbp) distributed across 16 chromosomes, for every two nucleotide substitutions accumulated on an individual chromosome, there is on average one expected mitotic crossing-over event on that chromosome and about 10% of possibility that one of those two chromosomal homologs will be lost. In *C. albicans*, the genome-wide single-nucleotide substitution rate was estimated at about 5.9 × 10^−10^ per base per generation; a mitotic crossing-over rate at about 10^-6^ – 10^-7^ per chromosome per division; and a whole chromosome loss rate of about 0.7 × 10^−6^ events per diploid genome per generation, with environmental conditions playing a significant role in the observed rates of chromosomal loss (Forche et al., 2011). Thus in *C. albicans*, the genome-wide nucleotide substitution rate was estimated to be about 3.5 times higher than that in *S. cerevisiae* while the mitotic crossing-over rate and whole chromosome loss rate were about ten times lower than those in *S. cerevisiae*. Comparisons between *C. albicans* and *S. cerevisiae* suggest that intra-strain heterozygosity would be higher in *C. albicans* than in *S. cerevisiae* in asexual populations of these two species, consistent with observations in natural strains (Peter et al., 2018; Wang et al., 2018). Together, the relative rates of nucleotide substitution to mitotic crossing-over and chromosomal losses that we assumed in **Figure 1** for *C. tropicalis* are within the range of those estimated for *S. cerevisiae* and *C. albicans*.

A slower rate of chromosomal loss relative to the rate of nucleotide substitution would result in a faster rate of accumulation of intra-strain heterozygosity and sequence divergence between all pairs of homologous chromosomes within each asexual lineage. In contrast, a faster rate of chromosome loss relative to nucleotide substitutions would result in progressive loss of heterozygosity within strains. Assuming the genome-wide nucleotide substitution rate in *C. tropicalis* was between those estimated for *C. albicans* and *S. cerevisiae*, we expect that it would take about 3,400 to 12,000 cell divisions to accumulate eight nucleotide substitutions on an average-sized chromosome of about 2 Mbp in *C. tropicalis* (14.6 Mbp/7 chromosomes). In an ideal lab environment on a rich agar medium where the cells replicate about once every four hours (Seervai et al., 2013), the number of cell divisions required to accumulate such mutations under the respective mutation rates of *C. albicans* and *S. cerevisiae* would correspond to between about 570 to 2,000 days. During this time, if we assume the chromosomal loss rate of *C. albicans*, there would be about 0.17% probability that one of the homologous chromosomes would be lost. However, if we assume the rate of *S. cerevisiae*, during the 2,000 days, there would be about 10.5% probability that one of the chromosomal homologs would be lost. In both scenarios, intra-genome heterozygosity could increase over time at the population level. Furthermore, using the mutation rates of *C. albicans* and *S. cerevisiae*, under ideal lab condition and assuming no chromosomal loss, it would take about 20,000 and 68,500 years respectively to accumulate 5% intragenomic heterozygosity, the average observed for the six putative hybrids of *C. tropicalis* by O’Brien et al. (2021). The introduction of chromosomal loss would result in the genome-wide heterozygosity ranges from 0% to close to 5% during this time period, with the variation of heterozygosity influenced by whether and when a chromosome homolog is lost. Even if we assumed one cell division per day for *C. tropicalis* in nature, using the mutation rates of *C. albicans* and *S. cerevisiae* and assuming no chromosomal loss, it would take about 120,000 years and 411,000 years respectively to reach the 5% intragenomic heterozygosity. Given the likely time of divergence between *C. tropicalis* and its closest known relative *Candida sojae* at about 10 million years ago (Gabaldón et al., 2016), the observed sequence divergence of ~5% between homologous chromosomes within each of the six putative hybrids of *C. tropicalis* could be reasonably accumulated through asexual reproduction, without invoking hybridization. Furthermore, if the *C. tropicalis* strains had defect(s) in DNA mismatch repair (i.e., mutator strains), such as those reported for *Cryptococcus deuterogattii* (Billmyre et al., 2017) and *Hanseniaspora* (Steenwyk et al., 2019), either transiently or long-term, the rate of mutation accumulation and sequence divergence between homologous chromosomes within such strains and clonal lineages could be significantly accelerated.

It should be noted that without chromosomal loss, mitotic crossing-over would not cause any change in intragenomic heterozygosity. However, in combination with chromosomal loss, mitotic crossing-over could change the distributions of heterozygosity along homologous chromosome pairs. The heterogeneous distributions of heterozygosity along most chromosomes of the model strain MYA-3404 are consistent with mitotic crossing-overs and chromosomal losses or BIR. Indeed, assuming asexual reproduction and one cell division per day over the 10 million years or so since the speciation of *C. tropicalis*, there would have been upward of ~18,250 (assuming the rate of *C. albicans*) or ~226,300 (assuming the rate in *S. cerevisiae*) mitotic crossing-overs for each homologous chromosome pair during the evolutionary history of each asexual lineage. Furthermore, assuming equal distribution of mitotic crossing-over events across each homologous chromosome pair of about 2 Mbp, such mitotic crossing-over numbers would represent approximately one crossing-over event between markers located every 9 bp to 110 bp on an average chromosome since speciation. Such a mean crossing-over distance could generate all the recombination events needed to produce the >n+1 inferred haplotypes as revealed for the *XYR1* and *MDR1* loci and for the observed phylogenetic incompatibility among the SNP sites within each of the six sequenced gene fragments for MLST by Jacobsen et al. (2008). Similarly, assuming one cell division per day over the 10 million years since the divergence between *C. tropicalis* and *C. sojae*, there would have been upward of 2,555 to 25,550 chromosomal loss events per chromosome for each asexual lineage from the present to when *C. tropicalis* diverged from its common ancestor with *C. sojae*. Depending on whether, where, and when the mitotic crossing-over events occur and whether and when chromosomal losses occur, a range of heterozygosity could be found among different parts of the same chromosome and among different chromosomes within most strains, consistent with what have been observed for the published genomes (Guin et al., 2020; O’Brien et al., 2021).

The above generalized model highlighted the possibility that asexual reproduction could explain the observed genetic and genomic variations in natural strains and populations of *C. tropicalis*. However, as indicated by others (Jacobsen et al., 2008; O’Brien et al., 2021) and in the sections above, sexual and/or parasexual reproduction could also generate the observed patterns. Distinguishing the modes of reproduction based on the observed signatures of recombination in *C. tropicalis* would require analyzing more strains and using different approaches. For example, signatures of sexual and/or parasexual reproduction would more likely be found by analyzing a larger number of strains with very high and very low intragenomic heterozygosity. Specifically, strains with lower intragenomic heterozygosity would allow relatively direct empirical identification of natural haplotypes. Those empirically identified haplotypes could be then compared with genome sequences of strains with high intragenomic heterozygosity to identify the haplotype relationships among strains, including any potential parent - “hybrid” offspring relationships and “hybrid-hybrid” sibling relationships. Such findings would provide empirical support the sexual/parasexual origins of the strains with high intragenomic heterozygosity.

## Conclusions and Perspectives

In this paper, I briefly reviewed our current understanding of the *C. tropicalis* genome and its population genetic variation, summarized the major expectations of the three different modes of reproduction (sexual, parasexual, and asexual), and compared the expectations of the different modes of reproduction with observed genetic variations. The comparisons showed that the traditional tests used to identify signatures of recombination (Hardy-Weinberg equilibrium, haplotype number > SNP number +1, phylogenetic incompatibility or the four-gamete test, and linkage equilibrium) are difficult to distinguish the three modes of reproduction. Furthermore, while parasexual and sexual reproductions can’t be excluded, asexual reproduction alone could produce all the observed population genetic variations in *C. tropicalis*, including the large variations in heterozygosity among chromosomes and chromosomal segments within individual strains as well as variations among strains.

Of special interest was the recent report of six (out of 77) natural isolates with high-level intragenomic heterozygosity, all of which were interpreted as putative hybrids between unknown parental strains (O’Brien et al., 2021). However, using mutation and chromosome loss rates data from *C. albicans* and *S. cerevisiae*, the analysis here showed that these isolates could have been derived from less than 10% of the *C. tropicalis* evolutionary history by reproducing only asexually to accumulate mutations between chromosome homologs with no or limited loss of heterozygosity. Indeed, the hypothesis that asexual reproduction alone without sexual/parasexual mating driving the observed genetic variations in *C. tropicalis* could be similarly applied to other organisms, including for explaining strains with significantly higher levels of heterozygosity than most other strains. At present, strains of human pathogenic yeasts with high levels of intragenomic heterozygosity are frequently considered as (putative) hybrids. The main evidence suggesting their hybrid origins have come from the sharing of putative haplotypes inferred based on whole-genome shotgun sequence data using phasing programs. At present, for all the inferred hybrids in the genus *Candida*, no parental strains have been unambiguously identified for any of the reported hybrids in the respective analyzed populations (e.g., Pryszcz et al., 2014; Wang et al., 2018; Mixão and Gabaldón, 2020; O’Brien et al., 2021). A key feature in haplotype phasing is to derive the minimal number of haplotypes capable of explaining the diploid SNP data (Scheet and Stephens, 2006). Thus, phasing programs tend to overestimate haplotype sharing among strains, especially in populations with small sample size, potentially resulting in false signatures of recombination and hybridization.

A clear exception to many of those putative hybrids is that of serotype AD strains of the human pathogenic basidiomycete yeast *Cryptococcus*. Within each serotype AD strains, allelic sequences identical to serotypes A and D sequences in the current natural populations have been found in the majority of loci that were individually cloned and sequenced, consistent with their recent hybrid origins of the majority of serotype AD strains (e.g., Xu et al., 2002; Xu and Mitchell, 2003). While phasing haplotypes from short-read genomic sequences of diploid strains with abundant heterozygosity represents a valuable approach for identifying potential hybrids and parasexual/sexual reproduction, single-molecule long-read sequencing or cloning and sequencing of individual alleles at many loci from a large number of *C. tropicalis* strains (including the putative hybrids) are needed in order to identify the correct haplotypes within individual strains. Such haplotypes would allow unambiguous inferences of phylogenetic relationships among alleles and chromosomal segments among strains. The unambiguous haplotype assignment and phylogenetic relationships inferred using such haplotype sequences are needed to exclude the possibility that asexual reproduction involving mutation accumulation, mitotic crossing-overs, and chromosomal losses might have generated the observed heterozygosity and allelic distributions within and among strains of *C. tropicalis*.

This review focused on the potential mode(s) of reproduction of *C. tropicalis* in nature and how the observed signatures of recombination in natural population of this species could be explained by asexual reproduction alone. Over the past three decades, signatures of recombination based on tests summarized in **Table 1** have been reported in most natural fungal populations examined so far, including those of saprophytic yeasts and molds, mushrooms, and plant, animal, and human fungal pathogens (e.g., Jacobsen et al., 2008; Xu et al., 2013; Taylor et al., 2015; Ashu et al., 2017; Peter et al., 2018; Wang et al., 2018; Xiong et al., 2021). So far, the inferred signatures of recombination have been almost exclusively attributed to sexual (and infrequently parasexual) reproduction. However, in the majority of fungi, their dominant mode of reproduction in nature is asexual, through mitosis and cell fission, budding, and/or hyphal extension (Xu, 2009; Taylor et al., 2015). As demonstrated previously (e.g., Forche et al., 2011; Zhu et al., 2014), recombination could occur during asexual reproduction in fungi in laboratory settings. If similar asexual recombination occurs in nature, abundant recombinants could also be produced in natural fungal populations during asexual reproduction, potentially generating observed genotypes and genotype frequencies similar to those expected from sexual (and parasexual) reproduction. The potential contribution of asexual recombination to genetic variations in natural fungal populations awaits critical investigation.

## Author Contributions

As the sole author of this manuscript, JX was responsible for conceptualizing, literature review, analyses, and writing this manuscript.

## Funding

This review was funded by the Natural Science and Engineering Research Council of Canada (grant number RGPIN-2020-05732), McMaster University (Global Science Initiative-2020-03), and the Institute of Bast Fiber Crops of the Chinese Academy of Agricultural Sciences during my visit.

## Conflict of Interest

The authors declares that the research was conducted in the absence of any commercial or financial relationships that could be construed as a potential conflict of interest.

## Publisher’s Note

All claims expressed in this article are solely those of the authors and do not necessarily represent those of their affiliated organizations, or those of the publisher, the editors and the reviewers. Any product that may be evaluated in this article, or claim that may be made by its manufacturer, is not guaranteed or endorsed by the publisher.
